# Recent advances in the management of pulmonary embolism: focus on the critically ill patients

**DOI:** 10.1186/s13613-016-0122-z

**Published:** 2016-03-03

**Authors:** Guy Meyer, Antoine Vieillard-Baron, Benjamin Planquette

**Affiliations:** Service de Pneumologie, Department of Respiratory Disease, Hôpital Européen Georges Pompidou, Assistance Publique Hôpitaux de Paris, 21 Rue Leblanc, 75015 Paris, France; Université Paris Descartes, Sorbonne Paris Cité, Paris, France; INSERM UMRS 970 and CIC 1418, Paris, France; Department of Intensive Care, Hôpital Ambroise Paré, Assistance Publique Hôpitaux de Paris, Boulogne-Billancourt, France; UFR des sciences de la santé Simone Veil, St Quentin en Yvelines, France; CESP, Equipe 5 (EpReC, Epidémiologie Rénale et Cardiovasculaire), INSERM U-1018, 94807 Villejuif, France; INSERM U UMRS 1140, Paris, France

**Keywords:** Pulmonary embolism, Right ventricle, Thrombolysis, Risk stratification

## Abstract

The aim of this narrative review is to summarize for intensivists or any physicians managing “severe” pulmonary embolism (PE) the main recent advances or recommendations in the care of patients including risk stratification, diagnostic algorithm, hemodynamic management in the intensive care unit (ICU), recent data regarding the use of thrombolytic treatment and retrievable vena cava filters and finally results of direct oral anticoagulants. Thanks to the improvements achieved in the risk stratification of patients with PE, a better therapeutic approach is now recommended from diagnosis algorithm and indication to admission in ICU to indication of thrombolysis and general hemodynamic support in patients with shock. Given at current dosage, thrombolytic therapy is associated with a reduction in the combined end-point of mortality and hemodynamic decompensation in patients with intermediate-risk PE, but this is obtained without a decrease in overall mortality and with a significant increase in major extracranial and intracranial bleeding. In patients with high-intermediate-risk PE, thrombolytic therapy should be given in case of hemodynamic worsening. Vena cava filters are of little help when anticoagulant treatment is not contraindicated, even in patients with PE and features of clinical severity. Finally, direct oral anticoagulants have been shown to be as effective as and safer than the combination of low molecular weight heparin and vitamin K antagonist(s) in patients with venous thromboembolism and low- to intermediate-risk PE.

## Background

Acute pulmonary embolism (PE) is a major cause of mortality. It has been estimated that over 370,000 deaths were related to PE in six countries of the European Union (with a total population of 454.4 million) in 2004 [1]. Several aspects of the disease have been investigated recently, and the results of these investigations have been associated with significant changes in the management of PE. The aim of this narrative review is to summarize for intensivists or any physicians managing “severe” PE the main recent advances or recommendations in the care of patients including risk stratification, diagnostic algorithm, hemodynamic management in the intensive care unit (ICU), recent data regarding the use of thrombolytic treatment and retrievable vena cava filters and finally results of direct oral anticoagulants. According to the risk stratification described below, critically ill patients include patients stratified as high risk or intermediate high risk.

### Risk stratification of patients with acute PE

Risk stratification allows physicians to deliver the optimal treatment to the patients according to the current knowledge, but another interest is to favor a quick triage of patients when they arrive at the hospital. PE has been shown to cover a wide spectrum of clinical severity with early mortality rates ranging between less than 1 % and above 50 % [2–4]. The case fatality rate of patients with sustained hypotension or cardiogenic shock, due to acute cor pulmonale (ACP), ranges from 35 to 58 %, and these patients are considered as high-risk patients [4–6].

Sustained hypotension, shock or even cardiac arrest is infrequent as over 95 % of patients with acute PE appear hemodynamically stable at presentation [4] and then will never be admitted to the ICU. However, many patients actually die before their admission, as shown in an old postmortem study reporting in 101 patients that near half of the patients died before admission in hospital within the first hour following onset of symptoms [7]. This can be considered as a limitation for risk stratification which is done in already selected patients, i.e., those who did not rapidly die and then were admitted to hospital.

A substantial body of evidence suggests that advanced risk stratification is able to distinguish between intermediate and low clinical risk in the normotensive patients.

Advanced age, major underlying conditions (cancer and cardiac or respiratory disease), clinical signs of right ventricular dysfunction (tachycardia and hypotension) and hypoxemia are the main clinical determinants of the outcome of patients with PE. This has been summarized by the pulmonary embolism severity index (PESI) and its simplified version (sPESI) (Table 1) [8, 9]. The principal strength of the PESI and sPESI lies in the reliable exclusion of an elevated risk for 30-day mortality (indicated by PESI classes I and II or by a sPESI < 1). On the other hand, in the absence of hypotension, these clinical rules have a low positive predictive value for the risk of death or PE-related complications [10].

**Table 1:** Simplified pulmonary embolism severity index, according to [9]

Variable	Points
Age >80 years	1
History of cancer	1
History of heart failure or chronic lung disease	1
Pulse rate ≥110 bpm	1
Systolic blood pressure <100 mmHg	1
Oxygen saturation <90 % on room air	1

Patients with none of the clinical variable (i.e., total score of 0) are considered as low risk and have mortality and pulmonary embolism-related complication rates significantly lower as those with a score of ≥1

 Right ventricular dysfunction (RVD) assessed by echocardiography or spiral computed tomography angiography and biomarkers including brain natriuretic peptide (BNP), N-terminal pro-BNP (NT-proBNP) and troponin, has been associated with an increased risk of death or PE-related complications including death due to PE, cardiogenic shock and recurrent PE in patients with normal blood pressure. In a cohort of 688 normotensive patients with acute PE, both NT-proBNP and echocardiography had a prognostic impact on top of that of the sPESI [11]. The risk of adverse outcome in patients with sPESI ≥ 1 was 2.5 % in those with normal value of NT-proBNP and normal echocardiography, 5.8 and 5.6 % in patients with either NT-proBNP ≥600 pg/mL or RVD on echocardiography, respectively. In patients with both elevated NT-proBNP and RVD, the risk of adverse outcome was 10.8 %, confirming that biomarkers and echocardiography have independent prognostic values [11]. In the PROTECT study, the combination of sPESI, troponin and BNP had a higher positive predictive value for a complicated course during follow-up than the sPESI alone or the combination of the sPESI with one of the biomarkers. In normotensive patients with sPESI ≥ 1, the risk of adverse event was 6.1 % in patients with normal values of biomarkers, 13.8 % in those with elevated BNP and 20.4 % in patients with both elevated BNP and troponin [12]. According to the recent guidelines of the European Society of Cardiology, patients with hypotension are defined as high-risk patients; among normotensive patients, those with a sPESI < 1 or PESI I-II are considered as low-risk patients without further risk stratification, those with a sPESI ≥ 1 or those with either RVD or elevated cardiac biomarkers are considered as intermediate-low-risk patients and those with sPESI ≥ 1 and both RVD and elevated cardiac biomarkers are considered as intermediate-high-risk patients [6].

### Diagnostic algorithm

Nothing is very new in this field, but it deserves a short comment. Recent guidelines of the European Society of Cardiology [6] re-emphasize that two different situations have to be differentiated.

The first one is a patient presenting with shock or hypotension and a clinical suspicion of PE. In the recent guidelines, hypotension is defined as a systolic blood pressure <90 mmHg, or a systolic pressure drop by ≥40 mmHg, for >15 min, if not caused by new-onset arrhythmia, hypovolemia or sepsis [6]. In this case, a rapid diagnosis is recommended by computed tomography pulmonary angiography (CTPA) if immediately available. If not, critical care echocardiography (CCE) is mandatory to quickly orientate the diagnosis and to look for signs of RV overload. It may rule out the diagnosis or confirm the high suspicion of PE if it demonstrates right ventricular dilatation, whereas many other causes in the ICU may induce RV dilatation, especially in ventilated patients. In the ICU, a patient is frequently admitted with shock without any clear suspicion of PE. By visualizing ACP, CCE helps intensivist to orientate the diagnosis. Some have even suggested emergency physicians to use CCE [13] and even very early in the pre-hospital setting, even though it cannot be currently recommended due to training issues. A special challenge for intensivist is intubated patients under mechanical ventilation (after cardiac arrest for instance). In this case, a transesophageal echocardiography may be performed to directly visualize thrombi in the main or right pulmonary arteries, avoiding transferring unstable patients for CTPA [14].

The second situation is a hemodynamically stable patient, with nor hypotension nor shock. Clinical probability of PE has then to be assessed by clinical judgment or predictive rule [6]. In case of high clinical probability, CTPA is recommended. Interestingly, CTPA may help risk stratification in the same time by evaluating RV size.

### The role of thrombolytic therapy

The role of thrombolytic therapy has been accurately defined for low-risk and high-risk patients with PE. In normotensive patients without signs of right ventricular dysfunction or damage, the risk of mortality and of PE-related complication is low and the use of thrombolytic treatment is not indicated, in part because of its associated bleeding risk [15]. Conversely, patients with high-risk PE have a high mortality risk when receiving anticoagulant treatment alone [6]. In this setting, the hemodynamic effects of thrombolytic treatment far outweigh its bleeding risk and the only contraindication to thrombolytic therapy in these patients is active uncontrollable bleeding [6]. In these patients, thrombolysis is associated with a reduction in mortality or recurrent PE, with a nonsignificant reduction in mortality [odds ratio (OR) 0.48, 95 % CI 0.20–1.15], a significant reduction in PE-related mortality (OR 0.15, 95 % CI 0.03–0.78) and a significant reduction of the end-point of death or treatment escalation (OR 0.18, 95 % CI 0.04–0.79) [15]. Noteworthy, only a minority of the patients included in these studies had systemic hypotension. Thus, guidelines recommend the use of thrombolytic therapy in clinically unstable patients with PE [6].

Until recently, the role of thrombolytic therapy was not accurately defined in the subgroup of patients with intermediate-risk PE. In the most comprehensive meta-analysis published before 2014, studies including clinically stable patients only (combining low-risk and intermediate-risk patients) were analyzed separately and did not demonstrate significant difference between thrombolysis and heparin alone for the risk of death (OR 1.16, 95 % CI 0.44–3.05) nor for the combined end-point of death or recurrent PE (OR 1.07, 95 % CI 0.50–2.30) [16]. Only two studies reported echocardiographic data with only 36 and 31 % of the patients having RVD [17, 18]. Biomarkers were not measured in any study. Thus, most of the patients included in these trials probably had low-risk PE and the role of thrombolytic therapy for patients with intermediate-risk PE cannot be defined on this basis.

More recently, the PEITHO study randomized 1006 patients with normal blood pressure and both RVD and elevated troponin to receive either heparin and tenecteplase or placebo and heparin [19]. The main clinical composite end-point of death from any cause or hemodynamic decompensation (or collapse) occurred in 13 patients (2.6 %) in the tenecteplase group and in 28 patients (5.6 %) in the placebo group (OR 0.44, 95 % CI 0.23–0.87, P = 0.02). This increase in efficacy was, however, obtained at the expense of an increase in major bleeding and intracranial bleedings. Major bleeding occurred in 58 patients (11.5 %) in the tenecteplase group and 12 patients (2.4 %) in the placebo group. Overall, 12 patients (2.4 %) in the tenecteplase group and 1 patient (0.2 %) in the placebo group had a stroke (P = 0.003). Mortality was 1.2 and 1.8 % in the tenecteplase and placebo groups, respectively (P = 0.42) [19].

A recent systematic review analyzed for the first time the results of thrombolytic therapy in patients with intermediate-risk PE [15]. In these patients, thrombolysis is associated with a nonsignificant reduction in overall mortality (OR 0.42, 95 % CI 0.17–1.03), with a significant reduction in PE-related death (OR 0.17, 95 % CI 0.05–0.67), a nonsignificant reduction in PE recurrence (OR 0.25, 95 % CI 0.06–1.03) but a significant increase in the risk of major bleeding (OR 2.91, 95 % CI 1.95–4.36) and fatal or intracranial hemorrhage (OR 3.18, 95 % CI 1.25–8.11) [15]. According to the recent guidelines from the European Society of Cardiology, the use of thrombolytic therapy is not recommended in all patients with intermediate-risk PE but should be considered if clinical signs of hemodynamic decompensation appear [6]. The guidelines suggest admitting these patients in the intensive care unit or an intermediate care unit for watchful waiting including cardiac monitoring and frequent clinical reassessment in order to provide secondary thrombolysis as soon as clinical signs of hemodynamic decompensation appear. According to recent data, this is the case of about 5 % of patients with intermediate-risk PE and hemodynamic decompensation occurs at a median delay of 1.8 days after admission (PEITHO).

 Using a reduced dosage of recombinant tissue-type plasminogen activator (rtPA), 50 mg for patients weighing ≥50 kg and 0.5 mg/kg for those weighing <50 kg, Sharifi et al. observed a significant reduction in the combined end-point of death plus recurrent PE with the use of rtPA (1.6 % in the thrombolytic group as compared to 10 % in the control group (P = 0.0489) [20]. In this rather small study, including 121 patients only, 61 receiving rtPA, the authors did not observe any major bleeding complication, suggesting that lower dosage of thrombolytic treatment may have a significant hemodynamic impact without increasing the risk of major bleeding. These results need, however, to be confirmed in a larger group of patients.

### Hemodynamic management in the ICU

**Fig. 1:**
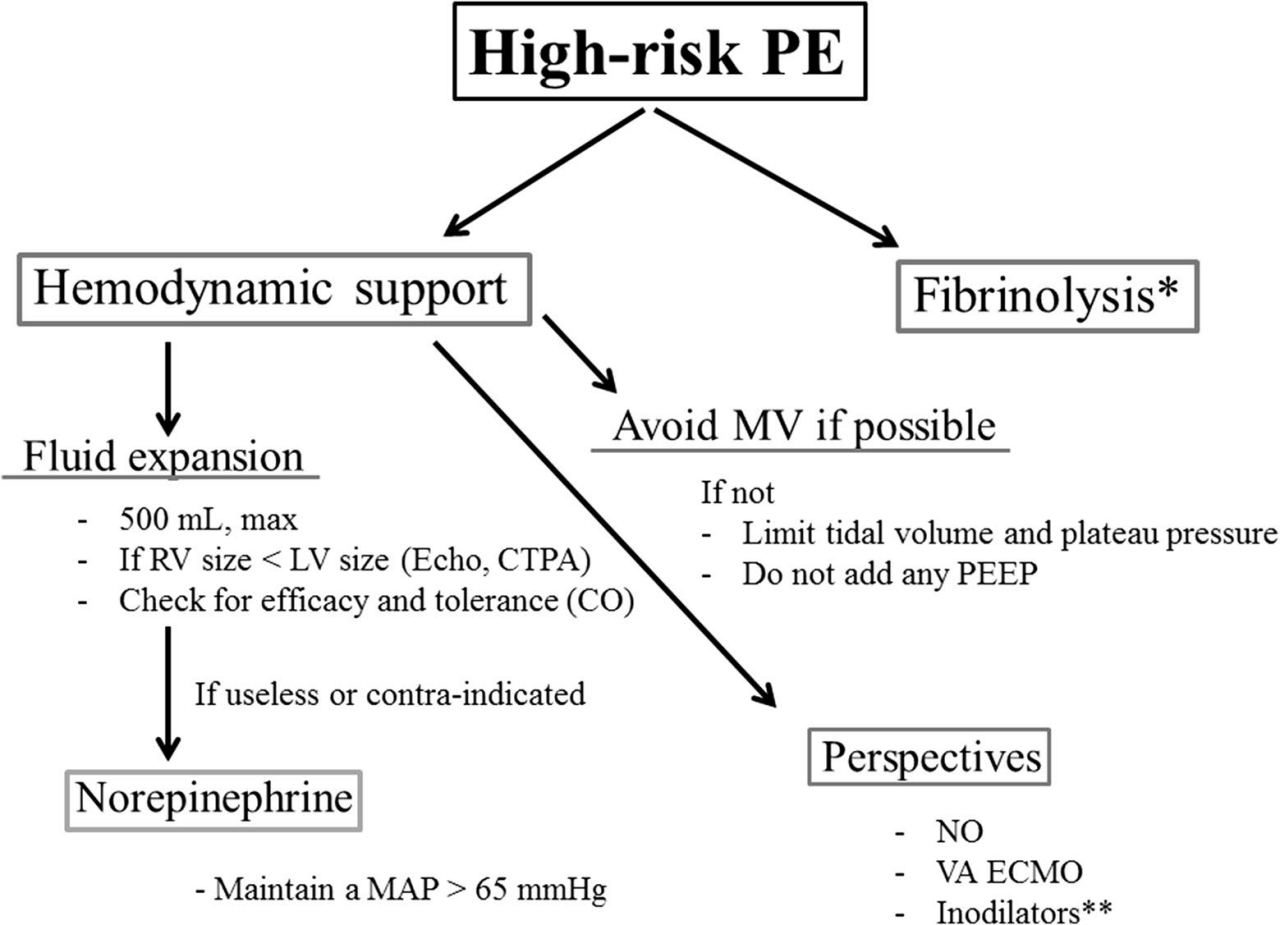
Proposal for hemodynamic management in high-risk PE: *in the absence of contraindication; **may improve the coupling between the right ventricle and the pulmonary circulation by increasing the RV contraction and decreasing the pulmonary vascular resistance. *RV* right ventricle, *LV* left ventricle, *CTPA* computed tomography pulmonary angiography, *CO* cardiac output, *MV* mechanical ventilation, *NO* nitric oxide inhalation, *VA ECMO* veno-arterial extracorporeal membrane oxygenation

Although data are lacking from clinical trials in humans, recent guidelines of the European Society of Cardiology recommended the following support: (1) to use volume expansion with caution, (2) to use norepinephrine infusion to improve RV function if necessary when blood pressure is low, (3) to ventilate patients, when required, with a low tidal volume and plateau pressure. A proposal for hemodynamic management is presented in Fig. 1. Historically, dobutamine was considered as the reference drug in case of hypotension/shock, although without strong evidence. Jardin et al. reported in a very small series of 10 patients spontaneously breathing that a 30-min dobutamine infusion (8.3 ± 2.7 µg/kg/min) significantly increases cardiac index and also reduces pulmonary vascular resistance [21]. Probably that the main interest of dobutamine compared to norepinephrine is that its infusion can easily done through a peripheral venous catheter.

One study performed in humans with intermediate-risk PE has reported that increase in cardiac output was inversely correlated with RV dilatation before fluid expansion [22]. The larger the right ventricle, the lower the positive effect on cardiac output and hemodynamics. Moreover, an experimental study in PE has shown that volume expansion could be deleterious by increasing RV stress and then decreasing cardiac output and blood pressure by its deleterious consequences on the left ventricle [23].

Conversely, rather than increasing RV overload by fluids, norepinephrine infusion has been reported as very efficient to support the right ventricle and to increase the cardiac output when the blood pressure is low [23, 24]. It especially acts by restoring the coronary perfusion pressure [25]. A study performed in a canine model of PE with shock has also reported that all dogs treated with norepinephrine were resuscitated and remained hemodynamically stable for 1 h, whereas all dogs treated with volume or isoproterenol died [26].

It is very unusual to have patients with PE under mechanical ventilation. It mainly occurs after cardiac arrest or for refractory shock. Positive pressure ventilation may be avoided when possible because it is deleterious by more increasing the RV afterload. If needed, it is recommended to limit the tidal volume and the plateau pressure.

A few treatments have been proposed but cannot be currently recommended due to the lack of data. In a few small series, nitric oxide inhalation has been reported to improve pulmonary function [27]. Finally, extracorporeal cardiopulmonary support may be an efficient rescue, as suggested in a porcine study but reported experience in humans is still lacking [28].

### What about vena cava filter?

The literature about vena cava filter is mainly based on cases series or even case reports and some case–control studies. Until recently, only one randomized controlled trial was available for the assessment of vena cava filters [29, 30]. In this trial, vena cava interruption using a definitive vena cava filter was associated with an early reduction in the risk of recurrent PE but with a late increase in recurrent deep vein thrombosis without significant difference in the risk of recurrent venous thromboembolism or death [29, 31]. Despite the paucity of prospective data, a dramatic increase in the use of vena cava filters has been reported recently, especially since retrievable filters have become widely available in the early 2000s. This is especially the case in the USA where about 12 and 9 % of patients with PE and deep vein thrombosis (DVT) received a filter in 2006 [32]. Conversely only about 2 % of all venous thromboembolism (VTE) patients underwent filter placement in a large European prospective registry [33].

The only indication for filter placement recommended by all guidelines is the contraindication to anticoagulant treatment in patients with PE or proximal DVT [30]. Although the use of vena cava filter has been advocated in patients with PE and high risk of recurrence, this has not been confirmed in the recent PREPIC II trial. A total of 399 patients with PE who have been selected for a high risk of death were randomized to receive anticoagulant treatment only or the combination of anticoagulant treatment and a retrievable vena cava filter which was retrieved at 3 months [3, 34]. The study did not demonstrate any significant difference in the main outcome of recurrent fatal or symptomatic nonfatal PE at 3 months between the groups (3.0 % in the filter group vs 1.5 % in the control group (RR 2.00, 95 % CI 0.51–7.89, P = 0.50). These results do not support the use of vena cava filter in patients with PE when anticoagulant treatment is not contraindicated.

### Direct oral anticoagulants for the initial treatment of PE

A series of new anticoagulant drugs have been developed recently. These drugs are direct inhibitors of factor Xa or factor IIa, and they are not subjected to food interaction and have minimal drug interactions. They can be administered orally at a fixed dosage without need for routine monitoring. Among these drugs, rivaroxaban, dabigatran, apixaban and edoxaban have been compared with the standard treatment of low molecular weight heparin (LMWH) and warfarin in patients with VTE including low-risk or intermediate-risk PE patients in large phase III trials [35–40]. A total of 27,023 patients with VTE were included in these studies; all studies included both patients with proximal DVT and PE except the Einstein-DVT study where only DVT patients were included and the Einstein-PE study where only patients with PE were included. The results of the six trials have been summarized in a meta-analysis concluding that direct oral anticoagulants (DOACs) nonsignificantly reduce recurrent VTE [relative risk (RR) 0.90, 95 % CI 0.77–1.06] and significantly reduce major bleedings (RR 0.61, 95 % CI 0.45–0.83), intracranial bleeding (RR 0.37, 95 % CI 0.21–0.68) and fatal bleeding (RR 0.36, 95 % CI 0.15–0.84) [41].

The patients with PE included in the six trials were analyzed separately and did not behave differently from the overall study population (RR for recurrent VTE: 0.89, 95 % CI 0.71–1.12) [41]. These drugs are now proposed as an alternative to the usual combination of LMWH overlapped and followed by vitamin K antagonist(s) (VKA) for the treatment of intermediate-risk or low-risk PE [6]. These drugs have not been evaluated in patients with high-risk PE who have been initially treated with thrombolytic treatment. In these patients, it should be wise to postpone the introduction of any oral anticoagulant after the patient has been stabilized with hemodynamic support and after the period of increased bleeding risk related to thrombolytic therapy which usually lasts 48–72 h.

### Mechanical approach for the treatment of PE

In a systematic review on mechanical or pharmaco-mechanical thrombectomy for PE, including 35 nonrandomized studies covering 594 patients, the rate of clinical success, defined as stabilization of hemodynamic parameters, resolution of hypoxia, and survival to discharge, was 87 % [42]. However, the contribution of the mechanical catheter intervention per se to clinical success is unclear because 67 % of patients also received adjunctive local thrombolysis. The reported rate of major complications including death from worsening RV failure, distal embolization, pulmonary artery perforation with lung hemorrhage, systemic bleeding complications, pericardial tamponade, heart block or bradycardia, hemolysis, contrast-induced nephropathy and puncture-related complications was 2 %, but publication bias probably resulted in underreporting of these complications [6]. While anticoagulation with heparin alone has little effect on improvement of RV size and performance within the first 24–48 h, the extent of early RV recovery after low-dose catheter-directed thrombolysis appears comparable to that after standard dose systemic thrombolysis. This has been shown in the only randomized controlled clinical trial performed about mechanical treatment of PE. The study included 59 intermediate-risk patients. When compared to treatment by heparin alone, catheter-directed ultrasound-accelerated thrombolysis—administering 10 mg t-PA per treated lung over 15 h—significantly reduced the RV/LV dimension ratio between baseline and 24-h follow-up without an increase in bleeding complications [43]. Although encouraging, these results should be considered as preliminary because the rather small size of the study precludes any meaningful estimation of safety and because the clinical consequences of the benefit observed regarding RV/LV diameter ratio remains unknown. In addition, this type of treatment is probably of little help in patients suffering from high-risk PE because of the prolonged treatment duration.

### Unsolved issues

Although this is not the primary scope of this review, some aspects of the recent risk stratification proposed by the European Society of Cardiology remain unclear. For instance, some patients considered as low risk according to the sPESI who should be considered for outpatient treatment already have increased biomarkers or right ventricular dilatation on computed tomography. Before specific outcome data are available, the most prudent option may be to admit these patients in the hospital.

In addition, a few therapeutic issues remain largely unsolved in patients with high-risk PE. The role of surgical embolectomy, if any, is unclear. It has been suggested without any evidence that surgical embolectomy could be more efficient in high-risk patients with mobile thrombi in the right cavities. In case of contraindications to thrombolysis, embolectomy could also be considered. If so, this should be based on a multidisciplinary approach and then low perioperative mortality, around 6 %, has been reported [44]. The management of the few patients with persisting cardiogenic shock after thrombolytic therapy also remains unclear. In this setting, surgical embolectomy is feasible without evidence of increased bleeding risk and has been associated with a better in-hospital course when compared to repeat thrombolysis in patients with massive PE who have not responded to thrombolysis [45, 46]. Finally, anecdotal evidence suggests that extracorporeal life support could be an option in these difficult cases or in patients presenting with cardiac arrest due to pulmonary embolism [47].

## Conclusion

Thanks to the improvements achieved in the risk stratification of patients with PE, a better therapeutic approach is now recommended from diagnosis algorithm and indication to admission in ICU to indication of thrombolysis and general hemodynamic support in patients with shock. Given at current dosage, thrombolytic therapy is associated with a reduction in the combined end-point of mortality and hemodynamic decompensation in patients with intermediate-risk PE, but this is obtained without a decrease in overall mortality and with a significant increase in major extracranial and intracranial bleeding. In patients with high-intermediate-risk PE, thrombolytic therapy should be given in case of hemodynamic worsening. Vena cava filters are of little help when anticoagulant treatment is not contraindicated, even in patients with PE and features of clinical severity. Finally, direct oral anticoagulants have been shown to be as effective as and safer than the combination of (low molecular weight) heparin and VKA in patients with VTE and low- to intermediate-risk PE.

## Abbreviations

ACP: acute cor pulmonale
BNP: brain natriuretic peptide
CCE: critical care echocardiography
CTPA: computed tomography pulmonary angiography
DVT: deep venous thrombosis
ICU: intensive care unit
LMWH: low molecular weight heparin
PE: pulmonary embolism
PESI: pulmonary embolism severity index
RVD: right ventricular dysfunction
rtPA: recombinant tissue-type plasminogen
sPESI: simplified version of PESI
VKA: vitamin K antagonist(s)
VTE: venous thromboembolism
